# Three-Dimensional Printing of Natural Materials Involving Loess-Based Composite Materials Designed for Ecofriendly Applications

**DOI:** 10.3390/ma14020293

**Published:** 2021-01-08

**Authors:** Hyunbae Lee, Jae-Hwan Kim, Seung-Muk Bae, Jiwon Oh, Heesu Hwang, Jin-Ha Hwang

**Affiliations:** 1 Department of Materials Science and Engineering, Hongik University, Seoul 04066, Korea; hyunbae90@naver.com (H.L.); jaehwan730@daum.net (J.-H.K.); jw3498@naver.com (J.O.); hshwang9797@gmail.com (H.H.); 2 Research Facility Center, Kunsan National University, Jeollabuk-Do 573-701, Korea; moki1492@kunsan.ac.kr

**Keywords:** 3D printing, loess-based materials, rapid setting/hardening, mechanical strength

## Abstract

In this work, loess-based materials were designed based on a multicomponent composite materials system for ecofriendly natural three-dimensional (3D) printing involving quick lime, gypsum, and water. The 3D printing process was monitored as a function of gypsum content; in terms of mechanical strength and electrical resistance, in the cube-shaped bulk form. After initial optimization, the 3D printing composition was refined to provide improved printability in a 3D printing system. The optimal 3D fabrication allowed for reproducible printing of rectangular columns and cubes. The development of 3D printing materials was scrutinized using a multitude of physicochemical probing tools, including X-ray diffraction for phase identification, impedance spectroscopy to monitor setting behaviors, and mercury intrusion porosimetry to extract the pore structure of loess-based composite materials. Additionally, the setting behavior in the loess-based composite materials was analyzed by investigating the formation of gypsum hydrates induced by chemical reaction between quick lime and water. This setting reaction provides reasonable mechanical strength that is sufficient to print loess-based pastes via 3D printing. Such mechanical strength allows utilization of robotic 3D printing applications that can be used to fabricate ecofriendly structures.

## 1. Introduction

The advent of the fourth industrial revolution has brought about advanced technologies such as artificial intelligence (AI), the Internet of Things (IoT), big data, and three-dimensional (3D) printing technologies. Rather than being independently positioned, these technologies are highly interconnected in a sophisticated manner. Since the pioneering introduction proposed by C.W. Hull [1,2], 3D printing has continually evolved with few limitations through the development of new materials and printing methodologies and using plastics, metals, glasses, and ceramics as materials and fused deposition modeling (FDM), stereolithography apparatus (SLA), selective laser sintering (SLS), and inkjet printing as processes [3,4,5,6]. Recently, there has been success in scaling 3D printing size with the help of either robotic systems or mechanical systems, which can expand the printing size beyond the traditional 30 cm × 30 cm. The synergic integration of 3D printing capabilities can open up new structural and/or aesthetic products with complex geometries. Applications are being expanded to new areas, including prototypes, art products, biomedical devices/assistants, architecture, and space residence/infrastructure [7,8,9,10,11,12]. 

Despite the advances in materials and processing, plastic materials suffer from the extreme environment encountered during 3D printing. These issues, including potential problems associated with VOCs (volatile organic compounds) and particles generated by 3D printers, have led researchers to search for new materials and novel 3D printing processes. Styrene and formaldehyde in ABS (acrylonitrile butadiene styrene), methyl methacrylate in PLA (polylactic acid), and caprolactam in nylon are typical materials that can cause potential negative health effects when used with 3D printers [13,14,15]. Higher printing temperatures can also produce a larger number of unhealthy particles. Research efforts have led to safer filament materials and atmospheric control that separates the 3D printing environment from the ambient one. Recently, cement-based materials and their applications have been combined with the 3D-printing concept to fabricate ecofriendly structural products [16,17,18,19,20]. However, there are no reports related to loess-based material, even though 3D printing was attempted in natural materials such as wood and bio-inspired ceramic materials [21,22]. Natural materials also represent a potential solution to problems originating from existing 3D printing materials. Among these, loess-based materials are well-known natural materials that have been employed worldwide in houses, in the form of small-sized structures. 

Impedance spectroscopy has been widely employed in cementitious materials since it was first introduced by McCarter et al. [23]. Alternating current (AC)-impedance characterization can resolve bulk information from electrode-associated phenomena. In particular, unlike electroceramics, cement-based material composites are dominated by the ionic conduction associated with the three-dimensional geometric interconnections [24,25]. These interconnections evolve continually through the formation of a resistive hydration product and narrowing of the ionic conduction pathway, leading to ever-increasing resistance and decreasing capacitance [25,26,27]. However, loess-based material composites have not been probed using impedance spectroscopy.

The current work focuses on the utilization and processing development of natural materials, which exist as clean and nontoxic constituents in natural ecology systems. Loess-based materials are selected as a model system for natural 3D printing. 3D printing products should be mechanically self-supporting with appropriate compressive strength resulting from their setting/hardening behaviors. The corresponding physical/chemical features are characterized using a multitude of probing tools, i.e., X-ray fluorescence (XRF) to determine the chemical composition, X-ray diffraction (XRD) for phase identification, and mercury intrusion porosimetry (MIP) for pore structure characterization. The loess-based materials are discussed in terms of use in nontoxic and ecofriendly 3D printing technologies. We also detail some remaining technical hurdles.

## 2. Materials and Methods

### 2.1. Design of Loess-Based Composites

Loess-based materials were chosen as a core material for natural 3D printing applications. The constituents are composed of high-quality loess materials (Dongbang Powtech Co., Gochang-gun, Korea), gypsum (Moongyo Gypsum & Engineering Co., Gimhae-si, Republic of Korea), and quick lime (Baekkwang Mineral Products Co., Ltd., Seoul, Republic of Korea). Before evaluating the properties of the loess-based composites, the constituent materials were analyzed to allow for effective material design. The microstructure and particle size were monitored using a scanning electron microscope (SU 8220, Hitachi, Japan) and particle size analyzer (LS 13320, Beckman Coulter, Inc., Brea, CA, USA). The crystal phases were analyzed by a high-precision X-ray diffractometer (Empyrean, PANalytical B.V, The Netherlands) with 2θ scans at 1.3 s/step using Cu Kα radiation (λ = 1.54 nm). The phase compositions were calculated using X’Pert HighScore Plus software based on Rietveld refinements. An X-ray fluorescence spectrometer (ZSX Primus II, Rigaku, Japan) was employed to analyze the material composition. Table 1 shows the composition zones of loess-based composites using loess, quick lime, and gypsum powders for two target systems: high viscosity for molding a cubic shape and low viscosity for 3D printing a beam shape. To fabricate cubic specimens, a certain combination of powders was mixed using an electric mixer, and the powders mixed with water to form a paste. The paste was poured into a 50 mm × 50 mm × 50 mm ceramic mold and cured at 60 °C under 95% relative humidity. The specimens were removed from the mold after 24 h. In the current work, 24 h was selected as a reference curing time for a comparison of 3D-printed products to the mechanical performance of cement-based materials.

Beam-shaped specimens were fabricated using our custom-made 3D printer. The paste with a low viscosity (with powder preparation the same as for the cubic specimen) was poured into a cylinder container and mechanically crushed to remove bubbles. The cylinder container was connected to a nozzle with a 4 mm diameter in a bottleneck-shaped structure for applying compaction pressure. Motorized pressure was applied to the cylinder container to discharge the loess composite from the nozzle. Rectangular column-shaped specimens of 15 mm × 15 mm × 245 mm were 3D printed and cured at 60 °C under 95% relative humidity. To monitor the setting/hydration state of the loess-based materials and cement-based materials, setting and hydration should be stopped properly. Therefore, the loess- and cement-based materials were subjected to a three-day solvent exchange process involving an organic solvent, i.e., acetone, and then dried in an electric oven for 24 h.

### 2.2. Mechanical/Electrical Analysis Preparation

The pore structure was determined by mercury intrusion porosimetry (AutoPore 9520, Micromeritics, Norcross, GA, USA), in which the specimen was prepared by stopping the hydration reactions using acetone and an aspirator. A pressure ranging from 3.64 × 10^3^ to 4.14 × 10^8^ Pa was applied to the pores, corresponding to a maximum and minimum pore size of 340 μm and 3 nm, respectively. The mechanical strength was evaluated by measuring the compressive strength of the cubic specimens at a pressing speed of 4 mm/min. Impedance spectra were acquired using an impedance analyzer (SI 1260, Solartron, UK) with an oscillating voltage of 0.1 V between 10 MHz and 1 Hz. A stainless steel plate was employed as an electrode, and the distance between electrodes was 40 mm. A three-point bending test was used on the 3D-printed, beam-shaped specimens to evaluate mechanical tensile properties using a custom-made universal test machine at a pressing speed of 0.1 mm/min.

## 3. Results and Discussion

### 3.1. Materials Analysis

**Figure 1:**
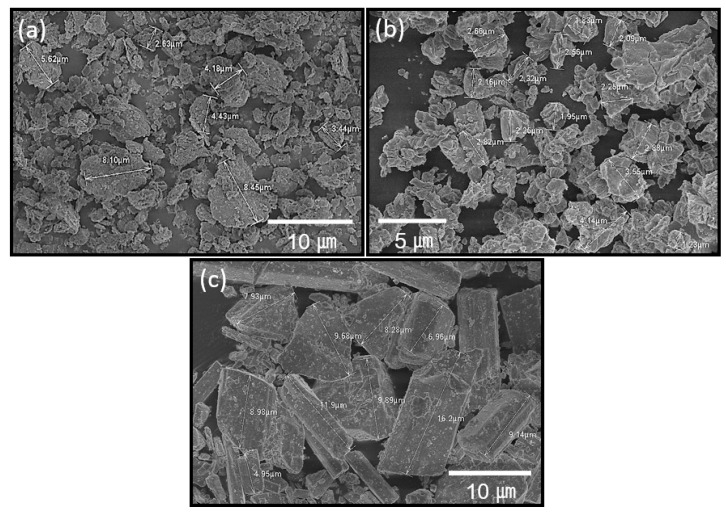
Field emission scanning electron microscopy images: (**a**) loess, (**b**) quick lime, and (**c**) gypsum.

Loess-based composite materials were designed considering chemical composition, particle size morphology, and particle size distribution. The chemical compositions are listed in Table 2; these were obtained via X-ray fluorescence analyses. In particular, the current loess materials include large fractions of Al_2_O_3_ and SiO_2_, i.e., 31.70 and 54.0 wt%, respectively, in addition to 9.93 wt% Fe_2_O_3_. Based on Table 2, the X-ray diffraction results indicate that the loess materials were made up of quartz, cronstedtite, hematite, and sodium dialuminum phyllo-decaoxodihydroxoalumotrisilicate. The quick lime was determined to be composed of lime, portlandite, and calcite, while the gypsum was bassanite. High-resolution electron microscopy images demonstrate irregular shapes in the loess and quick limes, as shown in Figure 1a,b. However, the gypsum is featured with angular polygons (see Figure 1c). The particle sizes of gypsum are larger than those of loess and quick lime, all of which have a critical influence on the rheology behaviors associated with 3D printing.

### 3.2. Mechanical Test

**Figure 2:**
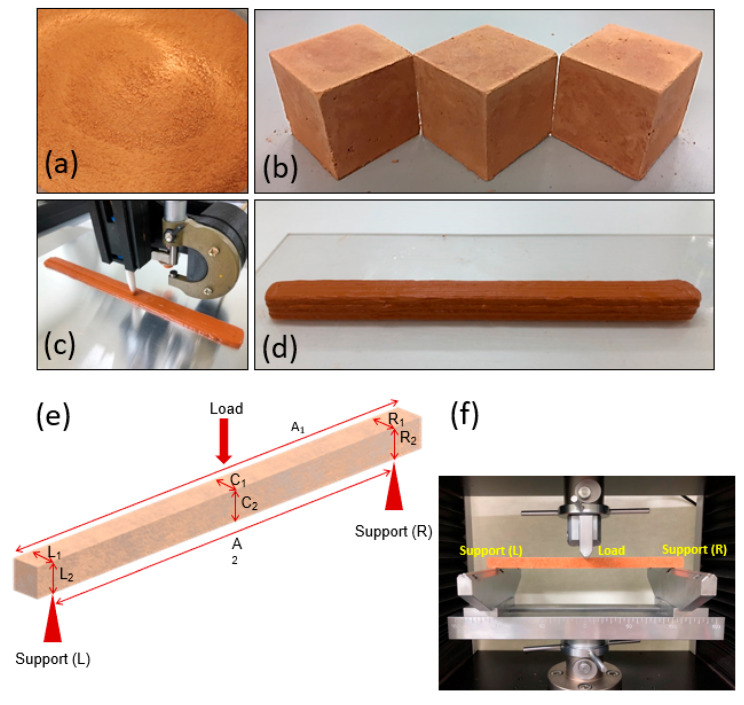
Specimen preparations for bulk composition and 3D printing applications: (**a**) powder mixture, (**b**) cubic specimen, (**c**) 3D printing process, and (**d**) 3D-printed specimen with a rectangular column shape. (**e**) Schematic sample geometry and (**f**) image of the actual three-point bending test of rectangular column specimens.

**Figure 3:**
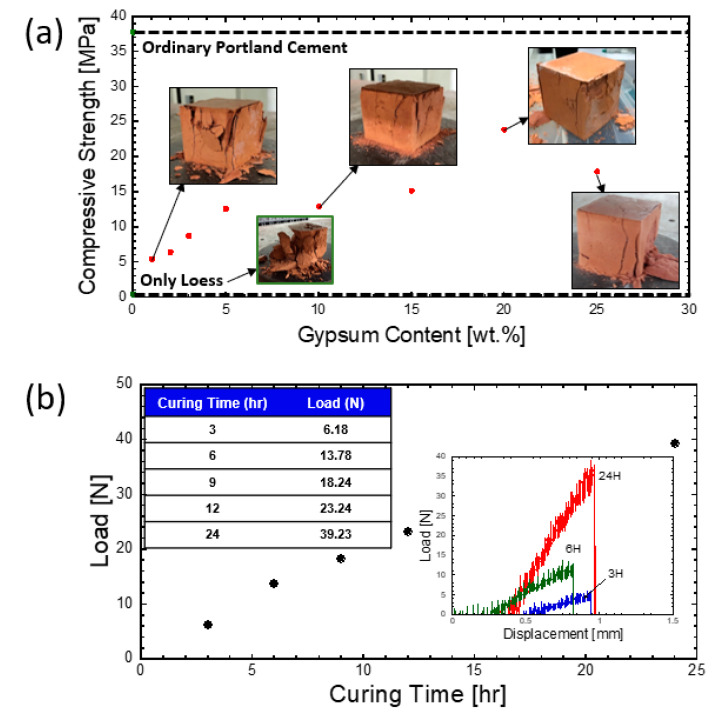
(**a**) Effect of gypsum content on compressive strength and (**b**) effect of curing time on a three-point bending test of a 3D-printed specimen (inset: load vs. displacement of the 3D-printed specimen).

Figure 2a,b shows the mixed powder and mortar specimens removed from a mold with dimensions of approximately 5 cm × 5 cm × 5 cm. After curing for one day, the fabricated loess-based bulk mixtures were subjected to the uniaxial compression test. The compressive strength is plotted as a function of gypsum content, which functions as a core binder. Loess-based composites showed lower strength as large-sized cubes of 5 cm × 5 cm × 5 cm. Thus, the 3D printing shape was a rectangular column with dimensions of approximately 1.5 cm × 1.5 cm × 24.5 cm. Figure 2c,d shows the corresponding 3D printing process and 3D-printed specimens. Figure 2e,f shows a schematic description of the sample geometry employed in three-point bending tests and an actual image of a specimen subjected to the three-point bending test. The 3D-printed column specimens were subjected to the three-point bending test in order for us to compare mechanical strengths. Without the presence of gypsum, the loess-based composite did not exhibit reasonable mechanical strength, as shown in Figure 3a. However, when gypsum was added to the loess-based composites, the mechanical strength reached an optimum of 23.9 MPa at 20 wt% gypsum. Interestingly, the optimized loess-based composite mixture (e.g., 20 and 25 wt% gypsum contents) showed highly reduced fragmentation compared to the high brittleness found in the loess-based composite without gypsum. Ordinary Portland cement was used as a reference system, where the water/cement (w/c) ratio was fixed to 0.40. The optimized loess-based mixture had 63% of the maximum strength of the cement paste. Figure 3b shows the curing time dependence of the 3D-printed loess-based composites; mechanical testing was performed using three-point bending tests. As shown in Figure 3b, the maximum load increased approximately linearly with curing time. The maximum mechanical load was 6.2 N after 3 h of curing and increased to 39.2 N after 24 h of curing. No plastic deformation was observed, and the samples only experienced elastic deformation following mechanical rupture.

**Figure 4:**
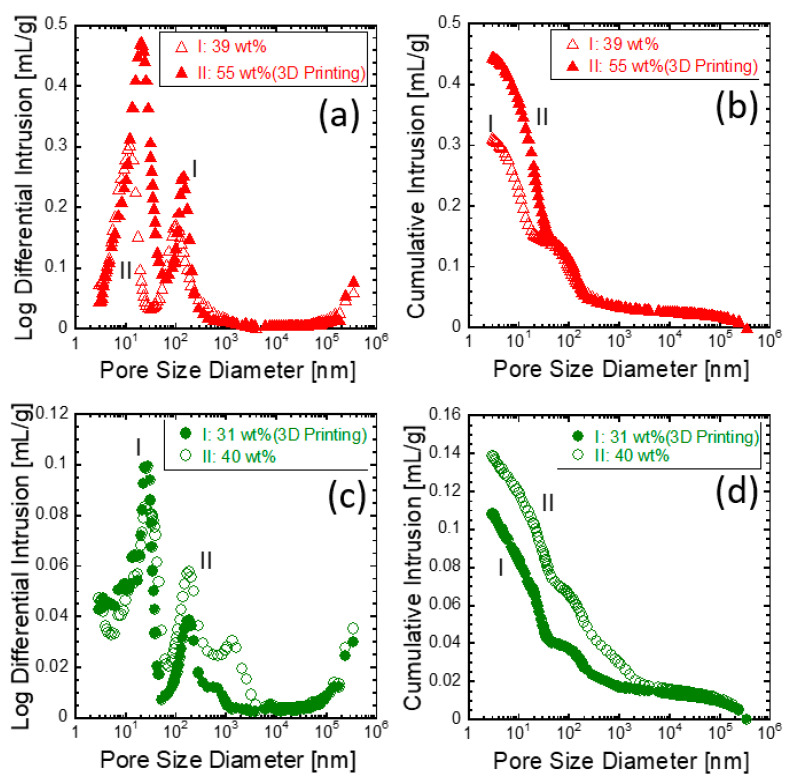
Mercury intrusion porosimetry results of (**a**,**b**) the loess-based composite and (**c**,**d**) ordinary Portland cement.

**Figure 5:**
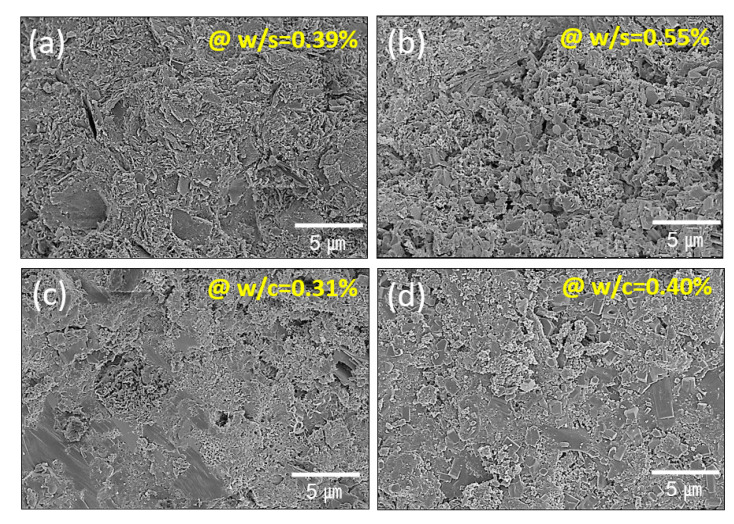
Field emission scanning electron microscopy images of (**a**,**b**) loess-based composites and (**c**,**d**) ordinary Portland cement; w: water, c: cement, and s: solid (loess + quick lime + gypsum).

Mercury intrusion porosimetry (MIP) has been used with cementitious and natural materials to extract quantitative data related to pore structures, where pore-based paths are interconnected in a highly tortuous, 3D manner (or with equivalently low interconnectivity) [28,29,30]. Furthermore, MIP characterization provides porosity values based on the amount of mercury that intrudes into the porous structures. MIP characterization was applied to the current loess-based composite system, as shown in Figure 4. Figure 4 shows the pore structure obtained from the loess-based materials in the form of either the bulk cube or 3D-printed rectangular column-shaped specimens; these are compared to cement-based materials as a reference system. The analyzed porosity information is summarized in Table 3. The loess-based materials exhibited 41.84% and 50.69% porosity for the bulk cube-shaped and 3D-printed rectangular specimens, respectively. However, the cement-based materials showed lower porosity of 19.94% and 24.43% for w/c = 0.31 and w/c = 0.4, respectively. As expected, a larger amount of water indicated a larger fraction of porosity. Furthermore, the cement-based specimens exhibited three peaks (or critical diameters) at 27.9, 181.2, and 553.2 nm and at 24.2, 181.4, and 1331.8 nm for w/c = 0.31 and 0.4, respectively, whereas the loess-based materials exhibited two peaks at 12.2 and 100.7 nm and at 20.1 and 139.4 nm for the bulk cube and 3D-printed rectangular column-shaped specimens. The high porosity of the loess-based composites is attributed to the water removal from the gypsum dehydrate and the remaining unreacted water. In the loess-based composites, the bulk cube-shaped specimen of Figure 5a exhibits a less open structure than that of the 3D-printed/hardened materials shown in Figure 5b. The cement-based materials of Figure 5c,d appear to be denser than those of Figure 5a,b. These observations were corroborated by the mercury intrusion porosimetry results shown in Figure 4, as mentioned above.

### 3.3. Electrical Test

**Figure 6:**
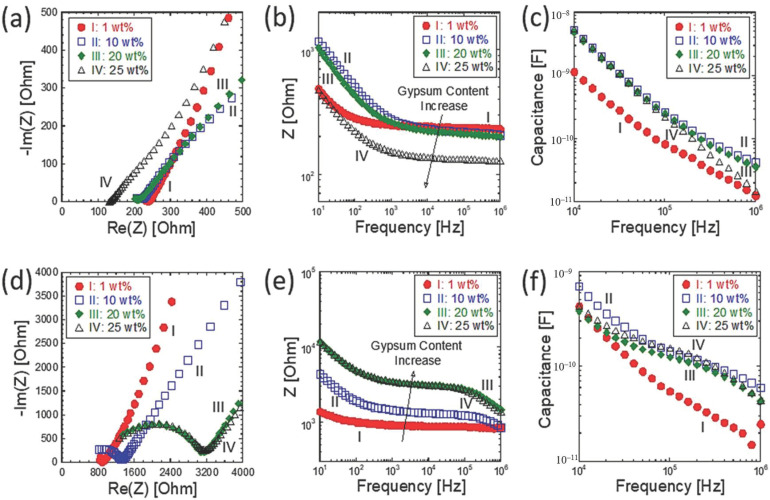
Effect of gypsum content on (**a**,**d**) impedance spectra, (**b**,**e**) |Z| vs. frequency, and (**c**,**f**) capacitance vs. frequency; (**a**–**c**) at 1 h and (**d**–**f**) at 24 h.

**Figure 7:**
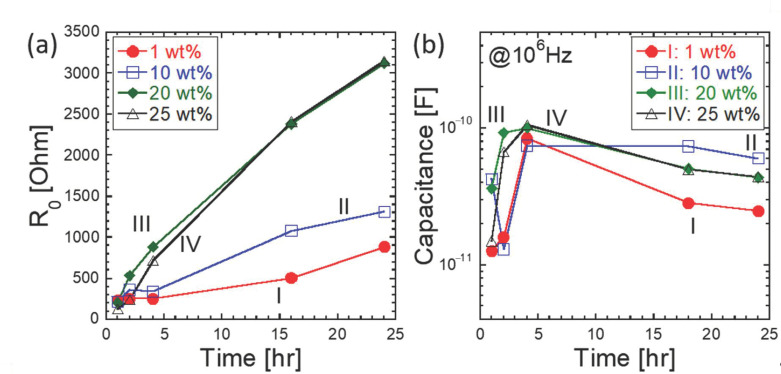
Effect of gypsum content on (**a**) bulk resistance and (**b**) capacitance (at 106 Hz) as a function of curing time.

Impedance spectroscopy was applied to the monitor setting (or hardening) in the loess-based composites. This was performed because impedance-based characterizations have been reported for hydration monitoring in cement-based materials [23,24,25,26,27]. In the cube-shaped specimens, the amount of gypsum was intentionally varied between 0 and 25 wt%. The corresponding impedance spectra are shown in Figure 6a,d at curing times of 1 and 24 h. Since the loess-based composites can be described as an ionic system, the impedance spectra are divided into high-frequency bulk responses and low-frequency, electrode-related responses; the former portion is controlled by ionic conduction through the 3D pore solution, and the latter portion is dominated by the charge transfer between the abovementioned ionic solution and the electronic electrodes. The corresponding impedance and capacitance Bode plots are shown in terms of absolute impedance and capacitance in Figure 6b,e and Figure 6c,f, respectively. At 1 h, the solution chemistry of the loess-based material composites was not stable, which may be due to an incomplete chemical equilibrium of the quick lime and gypsum; this indirectly indicates that ionic species continue to form in the loess-based materials. This belief is corroborated by the decreasing resistance observed upon the addition of gypsum. However, the capacitance reached a maximum value at 10 wt% gypsum and decreased with additional gypsum. At a curing time of 24 h, impedance increased with increasing gypsum, and the capacitance reached its maximum value at 10 wt% gypsum. The resultant bulk resistances are summarized in Figure 7a. The bulk resistance increased with longer curing times and increased significantly when gypsum was added to the loess-based materials. Initially, at around 1 h, the resistance values were similar, in the hundreds of ohms range. However, a longer curing time (e.g., 24 h) led to much higher resistance values, by at least 10-fold compared to the initial values. Interestingly, the capacitance exhibited two regimes before and after 5 h of curing, as shown in Figure 7b. At less than 5 h, the capacitance changed dramatically with time. However, after 5 h, the capacitance either decreased with curing time or reached a constant value.

### 3.4. Discussion

**Figure 8:**
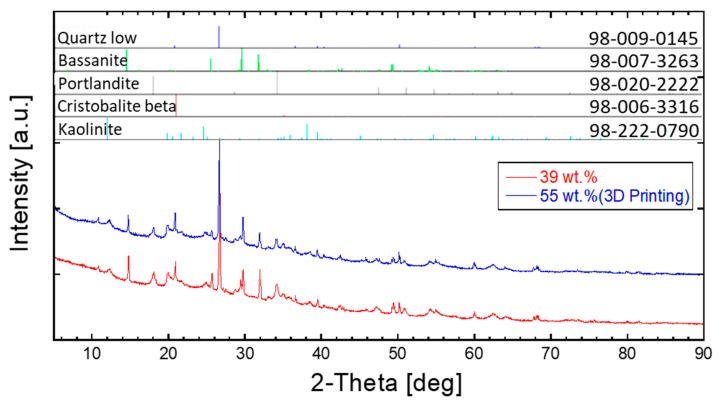
X-ray diffraction analysis of loess-based composites by water content (for bulk and 3D-printed loess-based composite materials).

**Figure 9:**
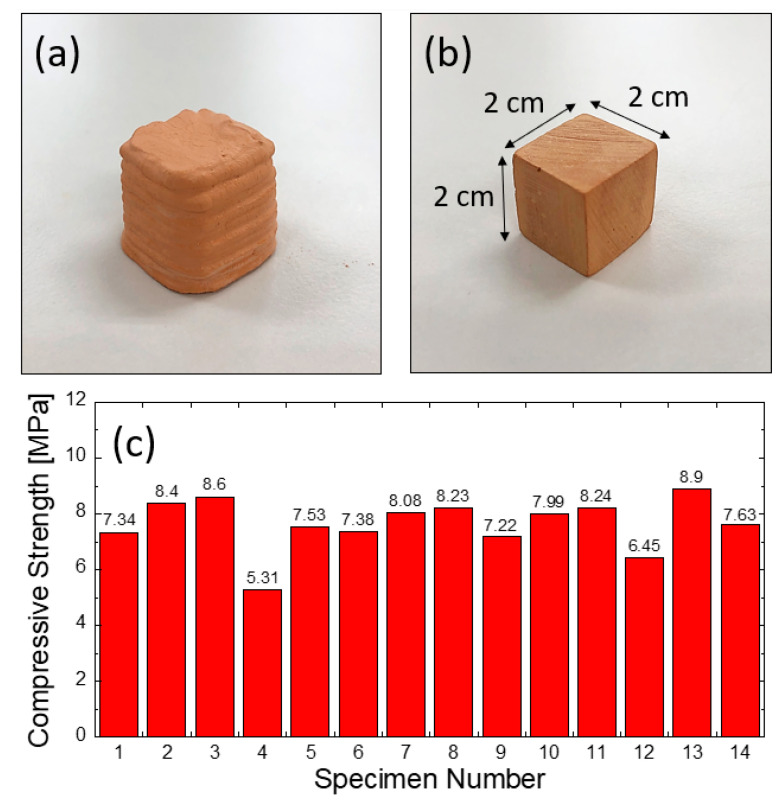
3D-printed cubes prepared through the customized 3D printing process (**a**) as 3D-printed and (**b**) polished cubes; (**c**) mechanical information obtained from 3D-printed cubes.

After setting, the corresponding mixtures were analyzed via high-sensitivity X-ray diffraction to determine the relationship between hardening and phase evolution in composite materials. The relevant X-ray information of the bulk and 3D-printed loess-based composite materials is shown in Figure 8, along with the constituent phases. The detected phases were low quartz (SiO_2_: 98-009-0145), bassanite (CaSO_4_0.5H_2_O: 98-007-3263), portlandite (Ca(OH)_2_: 98-020-2222), cristobalite beta (SiO_2_: 98-006-3316), and kaolinite (Al_2_SiO_5_(OH)_4_: 98-222-0790). Table 4 shows the phase information after hardening/setting, indicating large fractions of bassanite (CaSO_4_0.5H_2_O) and portlandite (Ca(OH)_2_). In addition, significant amounts of quartz and kaolinite were detected: 23.3 and 42.0 wt% for loess-based bulk mixtures and 31.8 and 50.7 wt% for 3D printing mixtures, respectively. The optimized composition mixture for 3D printing exhibited a much larger fraction of bassanite than portlandite, while the bulk mixture included similar amounts of bassanite and portlandite. Finally, as shown in Figure 9, the 3D printing process was optimized for cube-shaped specimens with dimensions of 2 cm × 2 cm × 2 cm. The average compression strength was 7.7 ± 0.93 MPa (see Figure 9c). Although the mechanical strength was lower than that of the bulk loess-based composite materials, it might be possible to further enhance its strength. The particle size distribution of loess is denoted as a single mode; however, gypsum and quick lime are characterized by two or three modes and have a dissimilar size range. The dissimilar particle size distribution should be considered in the future. The formation of bassanite in the loess-based composite materials contributes to their mechanical strength, allowing for loess-based 3D printing for ecofriendly and structural applications. Furthermore, the bassanite is believed to originate in the removal of water from gypsum dihydrate (calcium sulfate dihydrate) upon pore solution exchange involving organic solvents. Our future work will investigate refined setting chemistry and improvements in materials and processing in terms of strength and setting time, with the aim of optimizing the 3D printing capability on a larger scale.

## 4. Conclusions

In this work, loess-based materials were designed and optimized successfully in the forms of a cube and rectangular columns for ecofriendly applications using 3D printing. The feasibility of loess-based materials was evaluated in terms of mechanical, microstructural, and electrical factors. The realized 3D-printed products demonstrate adequate mechanical strength based on the experimental compressive strength of 7.7 MPa of the 2 cm × 2 cm × 2 cm cubes. This strength likely originates in the formation of gypsum hydrates. Impedance spectroscopy confirmed the formation of high-resistivity composites, leading to an increase in resistance. Mercury intrusion porosimetry characterization indicated that the porosity of loess-based composite materials was lower than that of conventional cement paste. Additionally, the critical diameters increased, which is related to the pore percolation pathway formed in 3D networks.

## Figures and Tables

**Table 1:** Composition design of loess-based composites for preliminary screening (referred to as the bulk format) and the 3D printing format.

Targeted System	Composition (wt.%)				Viscosity
	Loess	Quick Lime	Gypsum	Water	
Compressive Strength & Impedance Spectroscopy	55–79	20	1–25	39–45	High
3 Point Bending Test (3D printing)	55	20	25	55	Low

**Table 2:** Composition analyses of main constituents of the loess-based material composite for 3D printing applications; data were extracted from X-ray fluorescence analyses.

	Component	Na_2_O	MgO	Al_2_O_3_	SiO_2_	SO_3_	K_2_O	CaO	TiO_2_	Cr_2_O_3_	Fe_2_O_3_	P_2_O_5_	SrO
Material													
Loess		0.104	0.747	31.7	54.0	0.114	2.06	0.196	1.15	0.0266	9.93		
Quick Lime			0.812	0.0796	0.257	0.131		98.4			0.311	0.0205	
Gypsum		0.454			0.565	55.7	0.387	42.7					0.19

**Table 3:** Porosity of loess-based composite and ordinary Portland cement paste extracted from mercury intrusion porosimetry measurements.

Specimen	w/s = 0.39	w/s = 0.55	w/c = 0.31	w/c = 0.4
Porosity (%)	41.84	50.69	19.94	24.43

w: water, c: cement, and s: solid (loess + quick lime + gypsum).

**Table 4:** Phase composition analyses of loess-based composites based on quantitative X-ray diffraction.

Water (wt.%)	Quartz Low (SiO_2_)	Bassanite (CaSO_4_∙0.5H_2_O)	Portlandite (Ca(OH)_2_)	Cristobalite Beta (SiO_2_)	Kaolinite (Al_2_Si_2_O_5_(OH)_4_)
39	23.3	17.8	16.9	-	42.0
55	31.8	15.4	1.6	0.5	50.7

## Data Availability

The data of this work are available upon request to the corresponding author.
